# In search of features that constitute an “enriched environment” in humans: Associations between geographical properties and brain structure

**DOI:** 10.1038/s41598-017-12046-7

**Published:** 2017-09-20

**Authors:** Simone Kühn, Sandra Düzel, Peter Eibich, Christian Krekel, Henry Wüstemann, Jens Kolbe, Johan Martensson, Jan Goebel, Jürgen Gallinat, Gert G. Wagner, Ulman Lindenberger

**Affiliations:** 1 Max Planck Institute for Human Development, Center for Lifespan Psychology, Lentzeallee 94, 14195 Berlin, Germany; 2 University Clinic Hamburg-Eppendorf, Clinic and Policlinic for Psychiatry and Psychotherapy, Martinistraße 52, 20246 Hamburg, Germany; 3 German Institute for Economic Research, Mohrenstrasse 58, 10117 Berlin, Germany; 4 University of Oxfor The Health Economics Research Centre (HERC) Old Road Campus, Headington, Oxford, OX3 7LF UK; 5 Technical University Berlin, Institute for Economics and Business Law, Econometrics and Business Statistics, Straße des 17. Juni 135, 10623 Berlin, Germany; 6 Technical University Berlin, Institute for Landscape Architecture and Environmental Planning Landscape Economics, Straße des 17. Juni 145, 10623 Berlin, Germany; 7 Paris School of Ecoß 48 Boulevard Jourdan, 75014 Paris, France; 8 Centre for Economic Performance, London School of Economics, Houghton Street, London, WC2A 2AE UK; 9 European University Institute San Domenico, Fiesole, Italy

## Abstract

Enriched environments elicit brain plasticity in animals. In humans it is unclear which environment is enriching. Living in a city has been associated with increased amygdala activity in a stress paradigm, and being brought up in a city with increased pregenual anterior cingulate cortex (pACC) activity. We set out to identify geographical characteristics that constitute an enriched environment affecting the human brain. We used structural equation modelling on 341 older adults to establish three latent brain factors (amygdala, pACC and dorsolateral prefrontal cortex (DLPFC)) to test the effects of forest, urban green, water and wasteland around the home address. Our results reveal a significant positive association between the coverage of forest and amygdala integrity. We conclude that forests may have salutogenic effects on the integrity of the amygdala. Since cross-sectional data does not allow causal inference it could also be that individuals with high structural integrity choose to live closer to forest.

## Introduction

Research on brain plasticity supports the notion that our environment can shape brain structure as well as function^1,2^. In rodents experience-dependent alterations of the brain in the form of adult neurogenesis and synaptic plasticity has mostly been investigated using so-called “enriched environments”^3^. Donald Hebb in 1947 was one of the first to use an experimental paradigm comparing rats that could roam freely with those held in standard housing conditions^4^. These standard housing conditions usually consist of cages with access to food, water and bedding. The environmental enrichment condition in contrast varies between laboratories but usually comprises the provision of running wheels and toys and larger cages or rearing in larger groups of conspecifics^5,6^. Although the exact conditions are not yet defined key aspects that are repeatedly highlighted seem to be environmental complexity and novelty that offer a higher degree of stimulation, which in turn facilitates brain plasticity as revealed by histological studies measuring morphological features of neurons^7^. Unfortunately, it is unclear whether the typical laboratory housing conditions of animals that lack features of the natural habitat actually reflect a normal state or rather one of deprivation which is then compensated by the “enriched environment” manipulation^8^. Still the evidence acquired in studies on enriched environments in animals provides important information to infer what an enriched environment may look like in humans.

At first sight one may conclude that city dwellers experience more complexity and novelty in their environment compared to people living in more rural regions. However, in contrast to this, present research suggests that urbanicity encompasses a set of adverse psychosocial influences that facilitate chronic stress^6^. This is in line with epidemiological evidence showing that mental health problems are more frequent in urban as compared to rural areas. This has been shown for mood and anxiety disorders as well as schizophrenia, with up to 56% higher prevalence rates when comparing most to least urbanized regions^9,10^. Reasons for this may lie in the repeated infringement of personal space in cities that may trigger the brains’ threat system and in particular the repeated exposure to strangers may facilitate chronic engagement of the amygdala^11^.

At the same time a growing body of research has shown that living close to natural landscapes has beneficial effects on mental health^12,13^ as well as well-being, mood, cognition^14^, but also longevity^15^ and mortality^16^. First longitudinal studies have shown that moving to greener urban areas is associated with improvements in mental health supporting the causal interpretation of these previously reported effects^17^. Moreover, it has been shown that more green space in deprived urban neighbourhoods is associated with less perceived stress and healthier diurnal cortisol responses^18^. In Japan a so-called practice of “forest bathing” has been established under the term “Shinrin-yoku”. Although the empirical evidence base on Shinrin-yoku is small, first studies have demonstrated beneficial effects of passive viewing and active exploration of forest landscapes onto stress markers such as concentrations of cortisol, pulse rate, blood pressure, parasympathetic and sympathetic nerve activity^19,20^.

Although green landscapes and forests may be viewed as environmental enrichment factors in humans, its association with brain plasticity has to our knowledge never been investigated so far. What has been shown in the neuroscientific literature is that urban upbringing and city living might be detrimental by affecting stress processing in humans^6^. More concretely, current city living has been associated with increases in amygdala activity in comparison to living in more rural areas, whereas being brought up in an urban environment in the first 15 years of life increased stress related functional brain activity in the perigenual anterior cingular cortex (pACC)^21^. So it could be the case that an enriched environment is “overenriched”. More recently an exploratory study from the same research group reported an interaction between the neuropeptide S receptor gene, that has previously been associated with anxiety and stress phenotypes, and urban upbringing that modulates the amygdala response during stress exposure^22^. To our knowledge the only study so far that focussed on brain structure and environmental living conditions has associated urban upbringing in the first 15 years of life with reductions in grey matter volume in right dorsolateral prefrontal cortex (DLPFC) and the pACC (in men only) in a whole brain analysis^23^.

**Figure 1:**
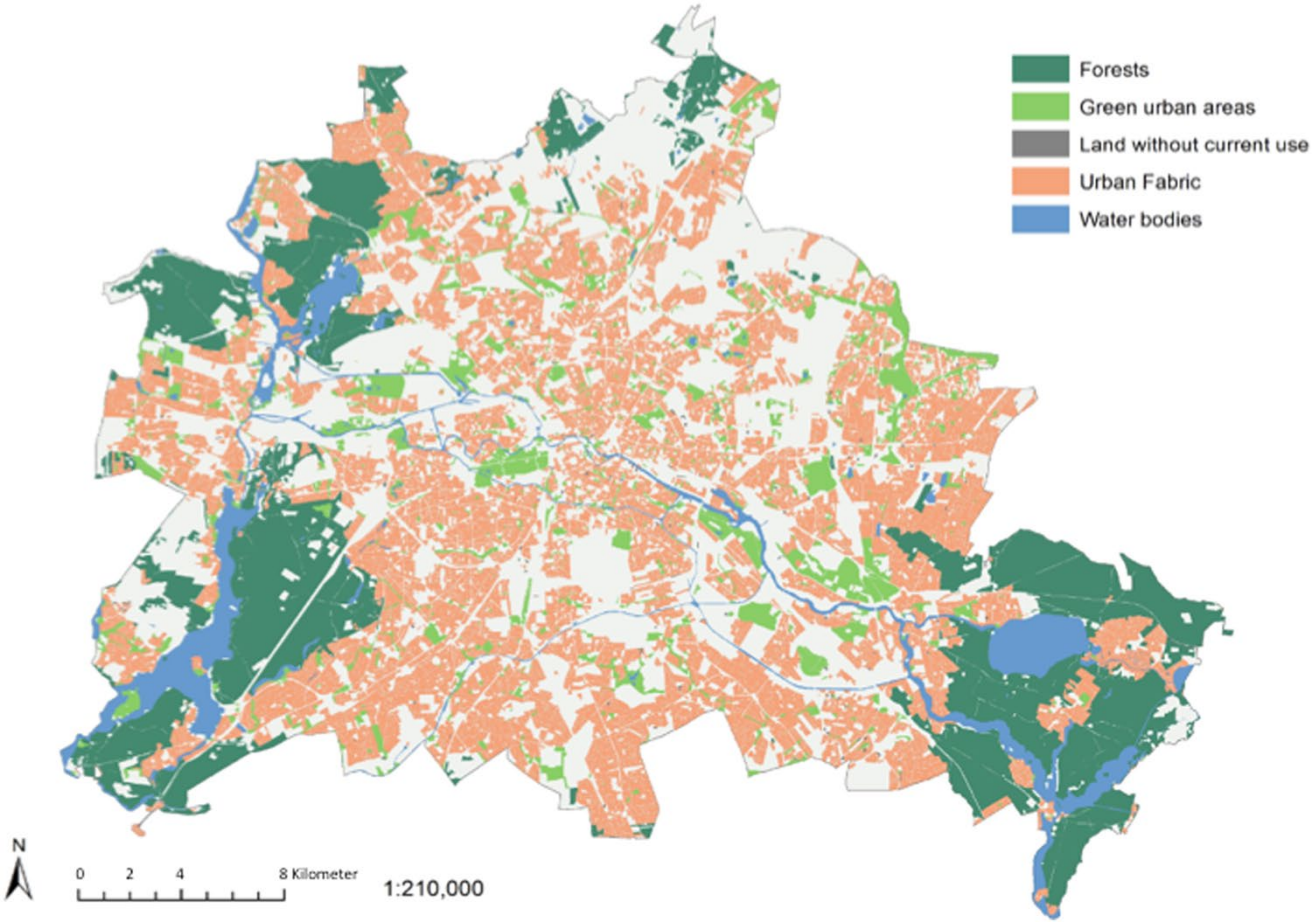
Spatial distribution of land use categories in the city of Berlin, Germany. Data taken from the Urban Atlas Land Use Data 2012 (European Environment Agency) created by means of the GIS software package Esri ArcGIS Desktop 10.3 (https://www.esri.de/support-de/produkte).

With the present study we set out to apply established characteristics of geographical features within cities in more depth and associate these cross-sectionally with brain structural integrity. Our aim was to investigate what may constitute an enriched environment for human beings on the micro level within the city of Berlin, complementing previous studies that discriminated brain differences of inhabitants of cities, towns and rural regions on the macro level. For this we used geocoded land use data of the neighbourhoods (Fig. 1) that our 341 elderly participants of the Berlin Aging Study II (https://www.base2.mpg.de/en,^24^) live in and associated this data with brain structural data of regions (amygdala, pACC and DLPFC) that have been implicated in prior studies comparing brain activity and brain structure in relation to living in cities or rural areas (Haddad *et al*.,^23^ Lederbogen *et al*.,^21^). We used a structural equation modelling approach to infer latent factors of brain integrity from three indicators originating from three different neuroimaging sequences (grey matter volume derived by means of voxel-based morphometry (VBM), mean diffusivity (MD) from a diffusion tensor imaging sequence, and magnetisation-transfer ratio (MTR)) for the regions of interest in amygdala, pACC and DLPFC (see^25^ for another example of a brain integrity factor comprising two neuroimaging modalities). This enables us to jointly incorporate information from these different brain imaging sequences.

## Results

On average the coverage of forest was 73252 m^2^ (SD = 238594), of water 62651 m^2^ (SD = 130393), of urban green 219817 m^2^ (SD = 179800), and of wasteland 7659 m^2^ (SD = 12247) within 1 km around the households of the included participants.

### Latent brain factors of structural integrity

Confirmatory factor analysis (CFA) was applied to define three latent factors capturing structural integrity of the amygdala, pACC and DLPFC. Each latent factor was defined by three indicators, namely the signal extracted from gray matter in VBM, MTR maps and MD maps (diffusion-tensor images) from each corresponding region. This saturated model perfectly reproduced the observed covariance matrix. Second we set up a model comprising all three brain factors and their between-factor covariances, where the indices indicated good model fit (RMSEA = 0.018, 90% confidence interval: 0.000–0.055, CFI = 0.999, AIC = 6031.04).

### Associations with geocoded land use variables

**Figure 2:**
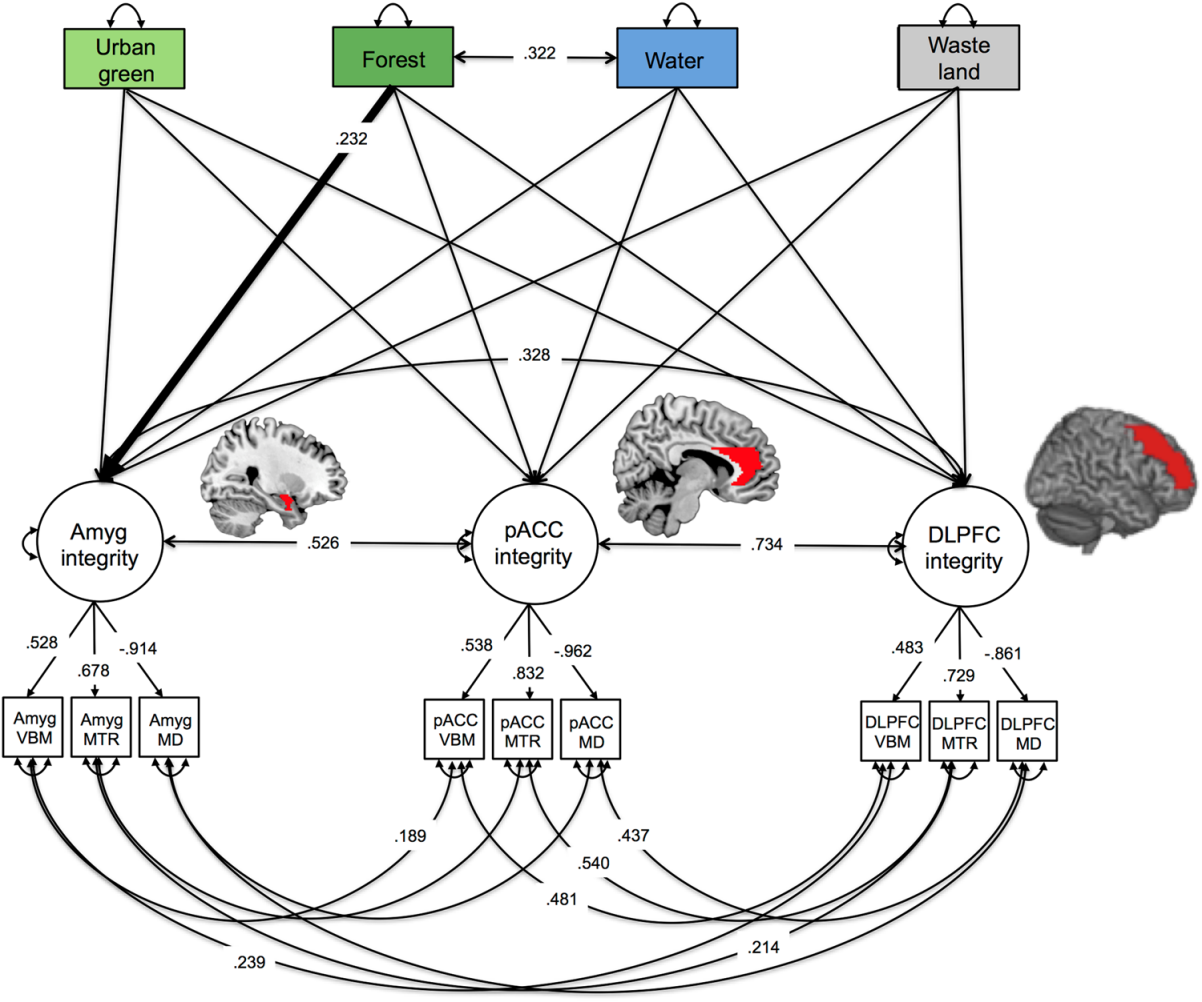
Depiction of the structural equation model with urban land use with in the radius of 1,000 metres around the individuals’ households. Squares represent observed variables and circles represent latent variables. Single headed arrows represent directional effects (the numbers next to it are the standardized regression coefficients), the single headed arrows from latent factors to manifest variables are an exception and represent factor loadings, double headed arrows between latent or manifest variables represent covariances between the variables and double-headed arrows with both heads pointing on a manifest variable represent the variance of a variable. All means were fixed to zero as the data were z-standardized. We report standardized estimates for estimates with *p* < 0.05. We controlled for age, sex and years of education, however these arrows are not included to increase readability of the graph. Abbreviations: Amyg: amygdala, pACC: perigenual anterior cingulate cortex, DLPFC: dorsolateral prefrontal cortex, VBM: grey matter probability extracted from voxel-based morphometry conducted on T1 weighted images; MTR: magnetisation transfer ratio; MD = mean diffusivity extracted from diffusion weighted imaging. The brain images depicted are based on the colin27 brain atlas^40^ and created using MRIcron (http://people.cas.sc.edu/rorden/mricron/install.html).

Then we set up a latent regression model to examine the associations between the three latent brain factors and our four geocoded land use variables, while controlling for age, sex and years of education. Geocoded land use variables were entered simultaneously as manifest predictor variables. Interestingly, the amount of forest within 1 km radius of the home address of the participants significantly predicted global amygdala integrity (β = 0.232, SE = 0.090, *p* = 0.010, Fig. 2). We obtained similar results when taking the amount of forest within a 500 m or 2 km radius into account (path between forest and amygdala 500 m: Wald χ2 (1) = 6.02, p = 0.014; 2 km: Wald χ2 (1) = 6.62, p = 0.010) but we decided to report the 1 km radius variable because it takes an average person 10–12 min to walk 1 km and therefore we would consider the area within 1 km around the house as the walking distance an older adult will very likely be able to cover.

Furthermore, a Wald test testing the present model against one where this path is equal to zero was significant (Wald χ2 (1) = 6.31, p = 0.012). All other paths between geocoded land use variables and brain factors were non-significant (β < 0.123, *p* > 0.084).

To exclude that the observed relationship between forest and amygdala integrity was driven by suppressor effects of other variables we estimated a model where we eliminated all brain regions except the amygdala and found the same significant association between the latent brain integrity factor and forest (β = 0.226, SE = 0.091, *p* = 0.013, Wald χ2 (1) = 6.00, p = 0.014). The same holds true when furthermore removing the other geocoded land use variables (urban green, water, waste land) (β = 0.230, SE = 0.081, *p* = 0.004, Wald χ2 (1) = 7.65, *p* = 0.006).

Moreover we tested whether living closer to forests is associated with wealthier residential areas by computing a correlation between the forest measure and the amount of pension annuity (*r* = 0.082, *p* = 0.25) of our participants, not finding a significant association. However, we reran the presented model while controlling for amount of pension annuity finding qualitatively the same results and a significant path from forest to amygdala integrity Wald χ2 (1) = 4.89,* p* = 0.027.

## Discussion

Within the scope of the present study we aimed at investigating potential geographical characteristics of the proximal living environment that may be considered features of an “enriched environment” in humans. Contrary to the intuition that city living per se would offer plenty of stimulation and therefore constitute an enriched environment with positive effects, previous research has demonstrated that psychiatric diseases are more common in urbanized as compared with rural regions^9,10^. Based on these findings neuroscientific evidence has been gathered implicating that current city living is associated with increased amygdala activity in a stress paradigm, whereas having been brought up in a city was associated with increased pACC activity, both reflecting a stronger response to stressful stimuli^21^. In terms of brain structure urban upbringing has been associated with reductions of grey matter volume in pACC and DLPFC (in men)^23^. Informed by these previous studies we set out to identify and characterize the geographical elements of a city that are associated with these brain structures following a suggestion by Kennedy and Adolph^26^ that studies should begin to derive recommendations for urban planning and architecture.

In a sample of older adults, all living in Berlin, we built latent factors each consisting of three different indicators of brain structural integrity (grey matter volume, magnetisation transfer ratio, mean diffusivity) per factor. This is an innovative approach that permits to integrate information from different brain imaging sequences, each providing distinct information on the integrity of the respective brain region. Each factor comprises an indicator of average probability of grey matter as derived from voxel-based morphometry, the average of the magnetisation transfer ratio map, which is used to evaluate macromolecular integrity and has been suggested as a marker for myelin in white^27^ but also in grey matter^28^ and the average of mean diffusivity measured by means of diffusion tensor imaging, which has been suggested to reflect the density of membranes and therewith represents barrier sparseness within brain tissue^29^ in the respective region. By establishing latent factors of brain integrity the dimensionality of the data can be reduced and the shared variance of the different sequences can be captured while measurement error is taken care of. We applied this procedure to the brain structures that were previously found to be associated with urban living as well as upbringing, namely amygdala, pACC and DLPFC. Then we tested the effects of urban green, forest, water and wasteland, assessed in terms of coverage within a circle with 1 km radius around the home address of the participants, onto structural integrity in the a priori defined brain regions. Our results reveal a significant positive association between the coverage of forest and amygdala integrity. Since the present data set is cross-sectional in nature and we cannot infer a causal directionality of the effects. However within the model we assumed that an effect from environmental variables onto brain structure is more likely than the reverse directionality where one would have to suggest that participants chose their geographical surrounding based on their brain structural integrity. In line with this assumed directionality, positive effects from enriched environments on morphological changes in the brain have been demonstrated in several animal studies^5,30,31^. Moreover a British longitudinal panel study has shown that over a period of five years those participants who relocated to a new residential area between the second and third year that was greener, mental health was significantly better in the post-move years^17^. Greater beneficial effects of local green space have been found in individuals that spend more time in their local neighbourhood (e.g. people not in work, older people), most likely due to the greater exposure to this green space^32^. This makes older adults a very good population to study relationships between environmental factors surrounding their home and health variables.

Remarkably our effect links the amount of forest in a 1 km radius to brain integrity, not urban green. The European Urban Atlas contains data of the year 2006, whereas the brain data has been acquired in 2014/2015 resulting in a relatively large period of time for potential land use changes. In comparison to forests urban green tends to change more quickly. This could potentially explain why forest, not urban green, is associated with brain integrity, despite many prior studies also reporting positive effects of urban green. In fact the total change of forest between the year 2006 and 2012 sums up to only 34,49 hectare while the total change of urban green within the same time period results in 7,74 hectare. Based on these data both land use categories appear as relatively persistent over time. The spatial distribution of land use within the city of Berlin shows, that forest sites are mainly located at the periphery of the city while urban green sites are equally distributed across the city area. Moreover, the average size of forest sites and urban green sites amounts to 44,48 and 5,04 hectares respectively. Due to location and comparable large sized forest sites are usually associated with lower levels of potential psychological stressors (e.g. traffic noise, air pollution and population density), that have been discussed in the literature (Tost *et al*. 2015). This could potentially explain our findings that forest, not urban green is associated with brain integrity.

However there is also data highlighting the potential salutogenic effects of forests. Recent neurobiological data using near infrared spectroscopy (NIRS) points at the prescinded role that the forest may play. Walking in a forest as well as sitting in a forest and watching lead to a reduction of prefrontal haemoglobin concentration, an observation that has been interpreted as a sign of relaxation^33^.

Above and beyond these previous studies we can characterize the association between structural brain integrity and different environmental parameters, not only forest, but also urban green, wasteland and water. Therewith our findings reach beyond the influential studies that have shown an association between brain function in a stress paradigm and brain structure and urban upbringing/ current living^21,23^. By exactly pinpointing which geographical features in the proximal participants’ habitat are associated with brain integrity we can start thinking about targeted city architecture to mitigate the typical urban effects on mental h﻿ealth. Data that is clearly needed in face of the fact that the landscape of human society is changing with an estimate that in 2050 almost 70% of the world’s population will live in urban regions (http://esa.un.org/unpd/wup/).

A major limitation of the present study is that our georeferencing data only assesses the environment 1 km around the place of residence of the participants. It does not allow us to infer where the participants actually spend most of their time. Due to the fact that almost all participants were retired, work-related commuting is unlikely to influence the results, however there might still be significant differences in the extent that participants actually spent time in and make use of their direct surrounding.

Another limitation of the study certainly is that the land use data used in this analysis refer to the year 2006, whereas the brain data has been acquired in 2014/2015. However, since the presence of the different land use categories is rather persistent over time the bias resulting from this time gap is negligible. Moreover, the land use data set applied to this analysis only contain areas with a minimum size of 0.25 hectare or 10 meter feature lengths neglecting spatial objects of smaller sizes and therewith smaller changes that may have occurred over time. The bias resulting from this measurement error is likely to be minor as forest sites and urban green sites should have a reasonable size to be effective.

Therewith, the results of our study may suggest that forests in and around the cities are a valuable resource that should be promoted. However future longitudinal studies are needed to investigate the causal directionality of the effect in order to disentangle whether more forest in ones habitat facilitates brain structural integrity or potentially those people with better brain structural integrity choose to live closer to forests. Moreover we need to investigate whether living close to the forest is associated with an absence of risk factors such as noise, air pollution or stress and thereby has beneficial effects or whether the forest itself constitutes a salutary factor that promotes well-being.

## Materials and Methods

### Participants and study design

Participants from the Berlin Aging Study II (BASE-II, for cohort characteristics and more see^24^ were recruited to take part in an imaging study.

After completion the comprehensive cognitive examination of BASE-II, eligible participants were invited to take part in one MRI session within a time window of 2–4 weeks after cognitive testing, consisting of 341 older adults aged 61–82 years (mean age 70.1, SD = 3.89; 131 female). On average the participants had 14.01 years of education (SD = 2.89) and a body mass index of 26.70 (SD = 3.51). Most of our participants were married and still living together (63%), while 14% were divorced, 4.4% single and 4.1% widowed. None of the participants took any medication that may have affected memory function or had a history of head injuries, medical (e.g., heart attack), neurological (e.g., epilepsy), or psychiatric disorders (e.g., depression). The different elements of the study were approved by the ethics committees of the Max Planck Institute for Human Development, the Charité University ethics committee and by the ethics committees of DGPs. Participants signed written informed consent and received monetary compensation for their participation in BASE-II and the MRI study. All experiments were performed in accordance with relevant guidelines and regulations.

### MRI acquisition

All images were acquired on a Siemens MAGNETOM Tim Trio 3 T scanner (Erlangen, Germany) using a 32-channel head coil. The T1 weighted images were obtained using a three-dimensional T1-weighted magnetization prepared gradient-echo sequence (MPRAGE) based on the ADNI protocol (www.adni-info.org) (repetition time (TR) = 2500 ms; echo time (TE) = 4.77 ms; TI = 1100 ms, acquisition matrix = 256 × 256 × 176, flip angle = 7°; 1 × 1 × 1 mm voxel size). Diffusion-weighted images were obtained with a single-shot diffusion-weighted spin-echo-refocused echo-planar imaging sequence (field of view (FOV) 218 mm × 218 mm; 128 × 128 matrix interpolated to 256 × 256; TE = 98 ms; TR = 11000 ms; 73 slices; slice thickness 1.7 mm; b-value 1000 s/mm2; 60 directions). Magnetisation transfer (MT) images were acquired consisting of two volumes acquired with identical settings (transversal, 256 × 256 pixels, TE = 5.5 ms, TR = 28ms 48 slices, voxel size 1mm × 1mm × 3mm). The first image (MT image) was acquired with a magnetic saturation pulse (1200 Hz off-resonance, 16 ms) and the second without (noMT image) a magnetic saturation pulse resulting in a proton-density-like image.

### Voxel-based morphometry (VBM) preprocessing

Structural data was processed by means of VBM8 (http://dbm.neuro.uni-jena.de/vbm.html) and SPM8 (http://www.fil.ion.ucl.ac.uk/spm) using default parameters. VBM8 involves bias correction, tissue classification and affine registration. The affine registered GM and white matter (WM) segmentations were used to build a customized DARTEL (diffeomorphic anatomical registration through exponentiated lie algebra) template. Then warped GM and WM segments were created. Modulation with Jacobian determinants was applied in order to preserve the volume of a particular tissue within a voxel leading to a measure of volume of GM.

### Diffusion tensor imaging preprocessing

Diffusion-weighted images were pre-processed using the FSL software package^34^, version 5.0. This included corrections of potential head movement and inspection of image quality. The first non-diffusion weighted image of each individual image set was used as a brain mask. The difference in alignment between this initial image and recurrent ones in the sequence was estimated using FLIRT^35^ and then corrected for by means of re-alignment. The resulting data was then processed via dtifit to obtain a measure of mean diffusivity (MD).

### Magnetisation transfer preprocessing

The magnetization transfer ratio (MTR) maps for each subject were calculated on a voxel-by-voxel basis according to the formula MTR = (noMT-MT)/noMT. Then the data was normalized into MNI space.

### ROI extraction

Based on prior studies exploring differences between brain function in participants currently living in cities compared to more rural residents we focussed on bilateral amygdala, pACC^21^ and DLPFC^23^ as defined by the automated anatomical labelling atlas^36^.

### Urban land use data

Within the BASE-II study we merged data assessed within the georeferencing dataset from Berlin city and its surrounding together with MRI data. The georeferencing data used was taken from the European Urban Atlas, provided by the European Environment Agency and constitutes a comprehensive and comparative cross-section study of urban land use in Europe, including data for major German cities^37^. The dataset contains polygon features representing real-world features characterised by areas. All areas greater than 0.25 hectare or 10 meter feature lengths for the reference year 2006 are assigned exclusively to well-defined land use categories. We included the categories “urban green”, since it has been shown to be associated with well-being^17,38^, additionally “forest”, and “water” since we thought, these categories could also be associated with positive effects on the brain, since these areas are frequently used for recreational purposes. “Wasteland” was included due to the fact that it is likewise a prominent category within Berlin. “Urban fabric” however, was not included, since it is a default category in urban regions, that does not reflect a specific land use category.

The category *urban green* is defined as land for predominantly recreational use including zoos, gardens, parks as well as suburban natural areas used as parks. Forests and other green fields are considered urban green areas in case there are traces of recreational use and they are surrounded by urban structures. Therefore forests within an urban setting as e.g. patches of parks canopied by trees fall into the category of urban green. Not included are, for example, private gardens within housing areas, cemeteries, agricultural areas, and other green fields not managed for recreational use. The category *forest* incorporates all (even privately owned) areas with ground coverage of tree canopy greater than 30% and tree height greater than five metres, including other kinds of vegetation at their borders, unless they are themselves part of green urban areas.

The category *water* incorporates lakes, rivers, canals exceeding one hectare. Notably, within parks, lakes are considered as waters and do not count to the green urban area surrounding them. Unlike the other land use categories all water bodies and water courses belong to the category *water* as long as they exceed an extend of 1 hectare.

The category *wasteland* (“*land without current use*”) is defined as areas in the vicinity of artificial surfaces still waiting to be used or re-used. The category contains waste land, removed former industry areas, gaps between new construction areas or leftover land in the urban context and is not used for recreation.

We defined the coverage urban land use, measured as the hectares covered by the land use category in a pre-defined radius of 1,000 metres around households, respectively.

### Structural equation modelling

We used structural equation modeling (SEM) to investigate the relationship between urban land use data and structural integrity, for two reasons. First, it enables us to take the information of three different structural brain imaging sequences into account to form a common latent variable capturing diverse information on the structural integrity of each region. This is sensible from a theoretical perspective as the sequences are designed to assess different aspects of brain integrity but when extracted from the same locations in space are positively inter-correlated. The advantage of this approach is that we can explore the brain data free from sequence specific and measurement error effects. A second reason is conceptual, as the model lets us formalize hypotheses about the relationships of the variables of interest. The focal question was whether geocoded land use variables quantifying characteristics of the environment surrounding the households of participants are specifically associated with the structural integrity of the amygdala, pACC and DLPFC. It should be noted that due to cross-sectional characteristic of this dataset we cannot clarify the causal direction of effects. However, we used SEM to define a model based on previously reported negative effects of urbanicity on amygdala, pACC^21^ and DLPFC^23^ where the four characteristics of urban land use predicted structural integrity of our three regions of interests.

Latent factor models were established by using MPlus v7 (Muthen LK, Muthen BO: Mplus User’s Guide. Los Angeles, Muthen & Muthen, 2010). To ease optimization, the data were z-standardized correcting for the extreme inter-variable differences in scale and location. We relied on standard indices such as the Root Mean Square Error of Approximation (RMSEA), the Comparative Fit Index (CFI), and the Akaike Information Criterion (AIC) for evaluation of model fit. Commonly accepted thresholds indicating good model fit are 0 <  = RMSEA <  = 0.05 and 0.97 <  = CFI <  = 1 and a lower AIC implies better fit^39^.

First we applied confirmatory factor analysis (CFA) to define three latent factors of individual differences in structural integrity of the amygdala, pACC and DLPFC. Each latent factor was defined by three indicators, namely the mean of the signal extracted from gray matter volume maps (VBM), magnetization transfer ratio maps and mean diffusivity maps (diffusion-tensor images) from each corresponding region.

### Data availability

The data can be requested from the first author S.K.
